# Expanding the genotypic and phenotypic spectra with a novel variant in the ciliopathy gene, *CFAP410*, associated with selective cone degeneration

**DOI:** 10.1080/13816810.2024.2369271

**Published:** 2024-09-04

**Authors:** Grace A. Borchert, Morag E. Shanks, Jennifer Whitfield, Penny Clouston, Shabnam Raji, Sian Sperring, Jennifer A. Thompson, Kanmin Xue, Samantha R. De Silva, Susan M. Downes, Robert E. MacLaren, Jasmina Cehajic-Kapetanovic

**Affiliations:** a Nuffield Laboratory of Ophthalmology, Nuffield Department of Clinical Neurosciences, University of Oxford, Oxford, UK; b Oxford Eye Hospital, Oxford University Hospitals NHS Foundation Trust, Oxford, UK; c Oxford Medical Genetics Laboratories, Oxford University Hospitals NHS Foundation Trust, Oxford, UK; d Australian Inherited Retinal Disease Registry and DNA Bank, Department of Medical Technology and Physics, Sir Charles Gairdner Hospital, Perth, Washington, Australia

**Keywords:** *CFAP410*, *C21orf2*, retinitis pigmentosa, skeletal abnormalities, cone dystrophy, ciliopathy, gene therapy

## Abstract

**Background:**

*CFAP410* (Cilia and Flagella Associated Protein 410) encodes a protein that has an important role in the development and function of cilia. In ophthalmology, pathogenic variants in *CFAP410* have been described in association with cone rod dystrophy, retinitis pigmentosa, with or without macular staphyloma, or with systemic abnormalities such as skeletal dysplasia and amyotrophic lateral sclerosis. Herein, we report a consanguineous family with a novel homozygous *CFAP410* c.335_346del variant with cone only degeneration and no systemic features.

**Methods:**

A retrospective analysis of ophthalmic history, examination, retinal imaging, electrophysiology and microperimetry was performed as well as genetic testing with *in silico* pathogenicity predictions and a literature review.

**Results:**

A systemically well 28-year-old female of Pakistani ethnicity with parental consanguinity and no relevant family history, presented with childhood-onset poor central vision and photophobia. Best-corrected visual acuity and colour vision were reduced (0.5 LogMAR, 6/17 Ishihara plates (right) and 0.6 LogMAR, 3/17 Ishihara plates (left). Fundus examination showed no pigmentary retinopathy, no macular staphyloma and autofluorescence was unremarkable. Optical coherence tomography showed subtle signs of intermittent disruption of the ellipsoid zone. Microperimetry demonstrated a reduction in central retinal sensitivity. Electrodiagnostic testing confirmed a reduction in cone-driven responses. Whole-genome sequencing identified an in-frame homozygous deletion of 12 base pairs at c.335_346del in *CFAP410*.

**Conclusions:**

The non-syndromic cone dystrophy phenotype reported herein expands the genotypic and phenotypic spectra of *CFAP410*-associated ciliopathies and highlights the need for light of potential future genetic therapies.

## 1. Background

Ciliopathies are a group of genetic conditions that disrupt the structure and function of cilia (1), and have a broad phenotypic spectrum involving brain, eye, bone, liver, kidney and reproductive organs. These conditions can be caused by pathogenic variants in many different genes. One of these genes is *CFAP410* (*Cilia and Flagella Associated Protein 410)*, (previously known as *C21orf2)*, which maps to chromosome 21q22.3 where pathogenic variants have most frequently been reported in association with rare syndromic ciliopathies involving the skeletal system (2,3). In particular, *CFAP410* is associated with Jeune Asphyxiating Thoracic Dystrophy (JATD) (2,4) and Axial Spondylometaphyseal Dysplasia (axial SMD) (5). It has also been described in association with amyotrophic lateral sclerosis (6).

In the eye, *CFAP410* encodes for a protein that localises to the primary cilium of photoreceptors. The CFAP410 protein is involved in ciliary maintenance, ciliary cargo transport in photoreceptors, and ciliogenesis. Pathogenic *CFAP410* variants reduce protein stability, impair cytoplasmic localisation and result in dysfunction of rod and cone photoreceptors (7). Previously, pathogenic *CFAP410* variants have been described with retinitis pigmentosa or cone rod dystrophy phenotypes, mostly in association with systemic skeletal abnormalities such as reduced stature, small and narrow thoraces, pelvic bone malformations, hip dysplasia, scoliosis, and platyspondyly (4,5,8,9). Herein, we report a novel variant in *CFAP410*, a homozygous in-frame deletion at c.335_346, associated with isolated cone dystrophy but with no systemic features, potentially expanding the genotypic and phenotypic heterogeneity of this rare ciliary gene.

## 2. Methods

A 28-year-old female patient was referred to the Oxford Eye Hospital. Data were collected as part of routine clinical care and collated retrospectively. At each visit, best-corrected visual acuity (BCVA) was measured and slit-lamp biomicroscopy and fundoscopy were performed. Multimodal retinal imaging included ultra-widefield scanning laser ophthalmoscopy (Optomap, Optos Inc, Dunfermline, UK), blue light fundus autofluorescence (BAF) and optical coherence tomography (OCT) (Spectralis, Heidelberg Engineering GmbH, Germany). ISCEV standard Full-field electroretinogram (ERG) and pattern ERG (PERG) were performed and electro-oculogram (EOG) was attempted. All ERG recordings were conducted with DTL fibre electrodes, and the impedance was <5kOhms and matched bilaterally. Pupils were dilated for the flash ERG and EOG. A repeatable PERG was measured. The data were compared to age-matched normative controls. Mesopic microperimetry (MAIA, Padua, Italy) was performed for each eye and compared to a reference database.

Molecular analysis of 111 genes for retinitis pigmentosa (RP) and RP-like phenotypes was undertaken by next-generation sequencing (NGS). A customised HaloPlex Target Enrichment system (Agilent Technologies) with Illumina MiSeq sequence technology was used. Data analysis was validated by an in-house analysis pipeline. Since the initial NGS testing did not detect a pathogenic variant, further sequencing was performed through the UKIRDC Project, followed by deep sequencing of achromatopsia-associated genes, *CNGA3, CNGB3, PDE6C* and *GNAT2*, and lastly whole-genome sequencing (WGS) (R32.2 Retinal disorders panel). Parental genetic testing was not possible.

The variant was classified in accordance with the American College of Medical Genetics (ACMG) and Association for Clinical Genomic Science (ACGS) guidelines (10). Information was gathered from the Leiden Open Variation Database (LOVD), the gnomAD/ExAC database, and Human Gene Mutation Database (HGMD). *In silico* predictions were performed using Mutation Taster, VEST INDEL, SIFT INDEL and SpliceAI (11–14).

## 3. Case presentation

**Figure 1.**
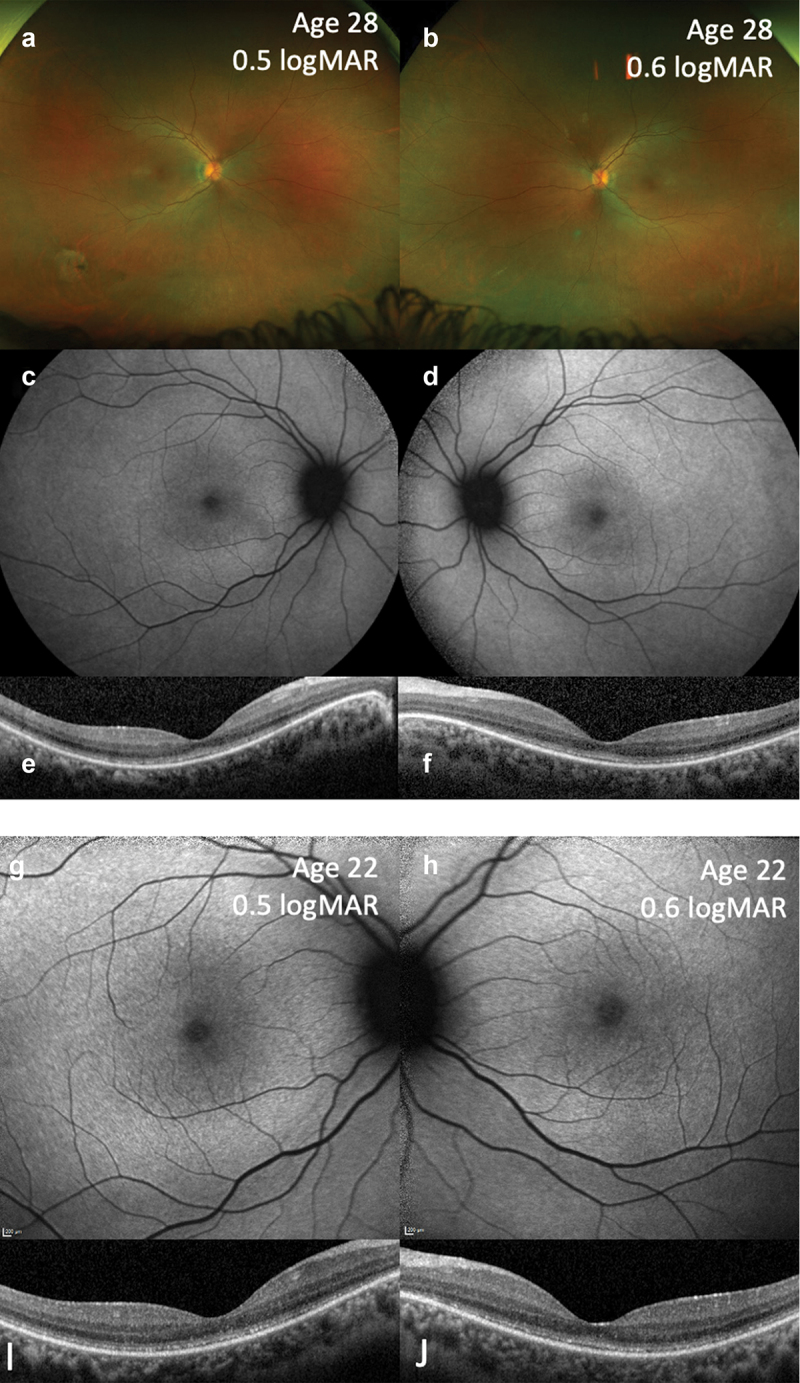
Pedigree of patient with *CFAP410* c.335_346del variant. Paternal and maternal grandmothers are third cousins.

A 28-year-old female, born to a consanguineous union of Pakistani descent, was referred to the ophthalmology genetics clinic with a history of poor vision since childhood (Figure 1). She described photophobia but had never reported difficulty seeing at night. There was no nystagmus or strabismus on clinical examination. BCVA were 6/18 right and 6/24 left eye. On Ishihara colour vision testing, 6/17 plates were seen by the right eye and 3/17 plates by the left eye. Anterior segments and fundus examination were normal with no pigmentary deposits in the retina.

**Figure 2.**
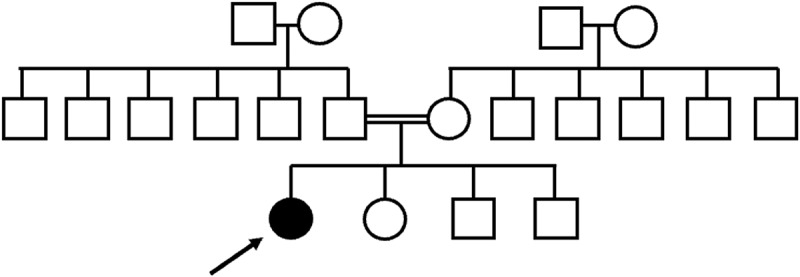
Multimodal imaging of the same patient at age 28 years (a-f) and 22 years (g-j) showed similar appearances, suggesting no or very slow disease progression. Optomap pseudocolour images at 28 years old (a, b), 55 degree BAF (c, d) and macular OCT (e, f), showing essentially normal fundal appearance and autofluorescence but mild disruption of the ellipsoid zone. Multimodal imaging at 22 years of age, 30 degree BAF (g, h) and macular OCT (i, j), with similar findings.

Optomap imaging confirmed no atrophy or peripheral pigmentation (Figure 2a,b). There were no marked changes on fundus autofluorescence imaging, and specifically no hyper-autofluorescent rings typically seen in retinal ciliopathies (6) (Figure 2c,d). OCT demonstrated disturbance and intermittent disruption of the ellipsoid zone (Figure 2e-f). The fundus autofluorescence (Figure 2g-h) and OCT images (Figure 2i-j) were unchanged from the previous examination 6 years prior to presentation, suggesting that there has been no obvious disease progression within this timeframe.

**Figure 3.**
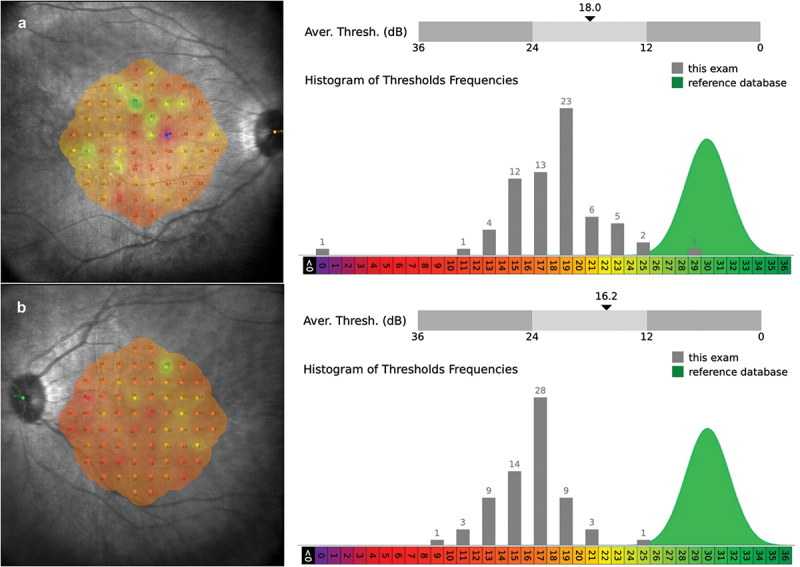
Microperimetry of the right (a) and left eye (b) Mesopic microperimetry showed reduced sensitivity compared to the reference database.

A differential diagnosis of achromatopsia, progressive cone dystrophy or occult macular dystrophy was made. Further phenotyping with electrodiagnostic testing demonstrated generalised cone dysfunction but normal rod responses on full-field ERG. In addition, 30 Hz cone flicker and S-cone responses were flat, consistent with bilateral generalized cone dysfunction, across all three cone types. The PERG was also completely extinguished. It was not possible to perform the EOG due to photophobia. Microperimetry demonstrated generalized reduced sensitivity compared to the reference database with an average threshold of 18 dB in the right eye and 16.2 dB in the left eye (Figure 3).

Since there is high prevalence of syndromic associations with CFAP410 and in particular the possibility of skeletal abnormalities, detailed history of musculo-skeletal system was undertaken with focused questions including history of joint pain, redness, swelling, deformities, stiffness, back and neck pain, bony fractures, gait disturbance, associated systemic features such as rashes, fevers, anorexia, weakness; none were identified. Hence, we had no clinical indication for skeletal imaging, although we acknowledge that this means that subclinical features of skeletal abnormalities cannot be excluded.

## 4. Genetic analysis and *in silico* pathogenicity predictions

**Figure 4.**
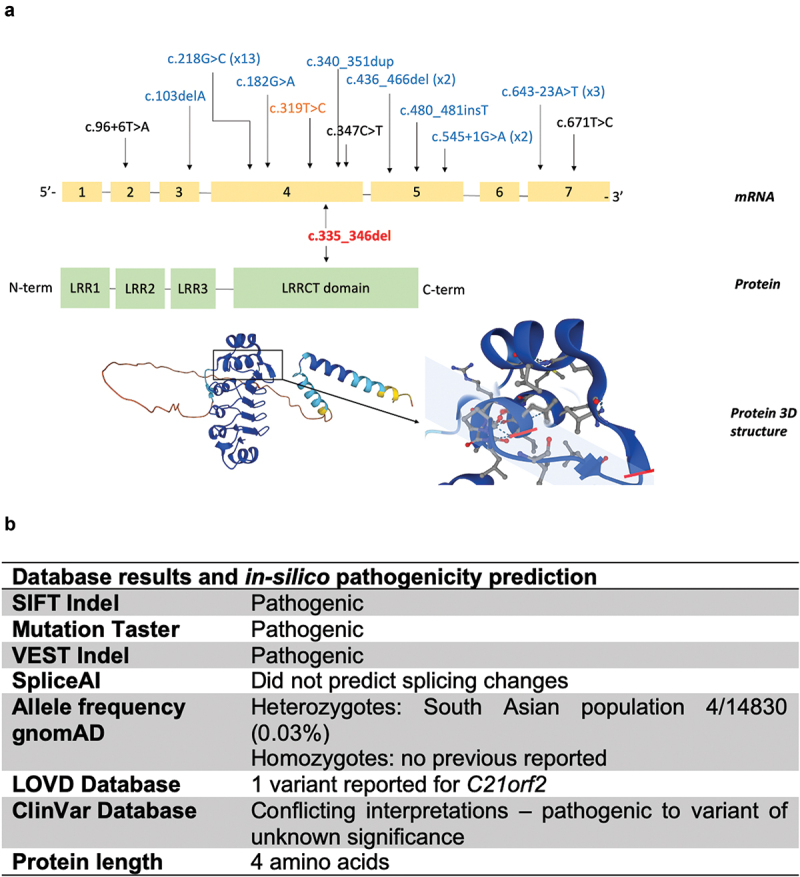
*CFAP410* mRNA, gene, and protein structure with database results and *in-silico* pathogenicity prediction. (a) *CFAP410* exons with previously described variants mapped and compared to our novel variant (bold, red). The *CFAP410* protein consists of 256 amino acid residues with known structural domains: 3 leucine rich repeats (LRR) and a downstream leucine-rich repeat C-terminal (LRRCT). The area of deletion from Leu112 to Leu115 is located within the LRRCT domain. In Homo sapiens, as predicted by AlphaFold, the region of interest is in the shaded area. (b) *In-silico* predictions and database information regarding the in-frame deletion.

Initial genetic testing with an NGS retinal panel did not detect any pathogenic variants. The patient was subsequently enrolled into the UKIRDC Project for enabling further genotyping using research tools, but no pathogenic variants were identified via these routes. Deep sequencing of achromatopsia-associated genes, *CNGA3*, *CNGB3*, *PDE6C* and *GNAT2* was also performed with no pathogenic yield. Finally, the patient was submitted for whole-genome sequencing (R32.2 Retinal disorders panel) once this was available as part of routine National Health Service (NHS) England for genetic testing, and a rare homozygous *CFAP410* variant, c.335_346del was identified (Figure 4). The genomic DNA coordinates of the variant on the GRCh38 reference genome spans from position 44333060 to 44333071 on chromosome 21. The variant represents an in-frame deletion encompassing positions 335 to 346 in the *CFAP410* coding sequence.

The ACMG and ACGS guidelines were followed for the initial genetic interpretation of the novel variant. The variant was detected at extremely low frequency) in the gnomAD/ExAC database. There have only been four heterozygous individuals (all in South Asian populations) and no homozygous individuals identified (constituting PM2 at supporting level of evidence). In the LOVD database, there were no *CFAP410* variants identified, although in the *C21orf2* Global Variome shared LOVD database, the same variant was reported in the homozygous state as a cause of autosomal recessive retinitis pigmentosa, although homozygosity was not confirmed in familial DNA samples (15). This was also reported in HGMD where there were several other cases in which *CFAP410* variants were considered causative for cone-rod dystrophy and syndromic cone-rod dystrophy with a short stature and narrow chest (8,16,17). However, in-frame insertions or duplications were considered causative for retinal dystrophy or cone-rod dystrophy, and a missense variant (c.331 G>A p.V111M in the next codon) was considered causative for RP and rod-cone dystrophy (3). In ClinVar, one case was retrieved where our identified variant was interpreted as pathogenic for retinitis pigmentosa and two cases where the variant was classified as variants of unknown significance.

Moreover, following the ACMG and ACGS guidelines, there was a moderate level of evidence of pathogenicity given that it is a 12 nucleotide in-frame deletion in a non-repeat region (constituting PM4). The variant is located near several other previously identified pathogenic variants; however, it cannot be concluded to satisfy PM1 criterion (occurring in a mutational hot spot and/or critical or well-established functional domain) because this criterion in the ACMG and ACGS guidelines are only applicable to missense variants and not in-frame deletions. For this reason, we classified the variant as being a variant of “uncertain significance” according to the ACMG/ACGS guidelines.

The novel variant was then further investigated and characterised (Figure 4a). The in-frame deletion c.335_346del occurs in the LRRCT region, a highly conserved protein motif. It removes four amino acids from the highly conserved consensus LRRCT sequence. The deletion of a portion of a highly conserved protein motif is predicted to have a significant functional consequence on the structure and function of the CFAP410 protein.

*In silico* analysis (Figure 4b) was performed to assess pathogenicity. Mutation Taster, VEST INDEL and SIFT INDEL predicted pathogenicity. Splice AI did not predict splicing changes.

A neighbouring pathogenic variant, a homozygous in-frame duplication of c.340_351dup shares similarities to the variant we describe. It is located near the boundary of exon 4, and the phenotype includes cone dysfunction with no obvious retinal pigmentation. However, it is described with rod involvement and a posterior pole staphyloma with a syndromic feature of short stature (16).

Further examining variants that lead to cone rod dystrophy in exon 4, there were 12 cone-rod dystrophies located in exon 4 out of all 18 cone-rod dystrophies (67%). These cases had additional ocular features such as a posterior staphyloma and pigmentation. In comparison, there were six cases of cone-rod dystrophies located outside of exon 4, which had RPE intermittent disruption and arteriolar attenuation (4).

The expert opinion at the UK National Clinical Genomics Multi-Disciplinary Team meeting also concluded that the patient’s phenotype and, in particular, the pattern of EZ disruption on OCT are consistent with cases that had been previously encountered with *CFAP410* variants affecting cones. The patient was referred for genetic counselling and continues to be monitored.

## 5. Discussion

We report the first case of isolated cone dystrophy in a 28-year-old female of Pakistani origin associated with a novel homozygous c.335_346del variant, in *CFAP410*. We report an isolated cone dystrophy with no systemic abnormalities.

The pathogenicity of this novel variant was further investigated and evaluated using with the available evidence. The variant in a homozygous state is predicted to affect the downstream LRRCT which is a highly conserved protein motif; it is a large in-frame deletion of 12 nucleotides and functional experimental results of nearby variants demonstrated pathogenicity. While these neighbouring variants were missense, insertions and duplications, it does suggest it is a critical domain. Further, *in silico* evidence suggests that the c.335_346del variant affects protein folding and hence optimal function. To date, 24 *CFAP410* variants have been reported in the literature in association with retinal dystrophy (1,2,5–9,16–19). There were 18 cases with a cone-rod dystrophy and 11 with retinitis pigmentosa out of a total 36 reported cases with a retinal phenotype, a majority have a skeletal phenotype; 5 cases had a posterior pole staphyloma and none have kidney involvement. Some cases were initially reported as isolated retinal disease in childhood (8) but were subsequently found to have skeletal abnormalities that have progressed to develop a hyperautofluorescent ring by early adulthood. In our case, there were no such features by the age of 28. One previously reported case was identified with a homozygous in-frame duplication (c.340_351dup) (8) in the same LRRCT domain of *CFAP410* close to the location of our variant, supporting our predictions regarding pathogenicity.

This case is associated with an in-frame deletion, thus a protein with a significant deleted region may impair the cilia-associated protein function and folding. Since parental genetic testing was not possible, there is no way to exclude uniparental disomy. However, the in-frame deletion may not have completely abolished protein function, as has been seen with hypomorphic variants or in-frame deletions in other genes associated with recessive disease. This could explain why the phenotype was not as severe compared to other *CFAP410* pathogenic variants as there were no systemic features, no hyperautofluorescent ring and no rod involvement, by the age of 28.

Functional experiments have demonstrated that Y107H, a relatively common pathogenic variant in *CFAP410*, can lead to decreased protein expression *in vitro* (20). There were decreased levels of CFAP410 and altered localisation of mutated protein products relative to wild type, suggesting that this may be a null variant (20). *CFAP410* has been shown to be expressed in the retina during the developmental stage and in the adult retina, the protein localises to the photoreceptor cilia2 (20).

Compared to other retinal ciliopathies, which typically affect both rods and cones, there was no observed hyperfluorescent ring in our case. The closest variant reported showed progression to a well-demarcated hyperautofluorescent ring over 10 years, suggesting it takes a significant time to develop (16). Moreover, a recent case series on *CFAP410*-related retinopathy (21) describes a double hyperautofluorescent ring in some patients. It has been suggested that the hyperautofluorescent ring arises following increased outer segment phagocytosis by RPE in patients with RP (22). A hyperautofluorescent ring has also been seen where there is preserved RPE and ellipsoid zone in patients with RPGR (23). In our variant phenotype, there were subtle signs of macular disturbance and intermittently disrupted ellipsoid zone on OCT suggesting cone-only involvement. An annulus of increased autofluorescence signal would have been expected to have developed by the age of 28, despite the mild phenotype. Either it will remain stable, or progress towards a cone-rod dystrophy with a hyperautofluorescent ring.

Genetic and vision restoration treatments are under development (24) and one gene therapy treatment, Luxturna, is currently approved for one specific type of inherited eye disease, the *RPE65*-related Leber congenital amaurosis. Currently, there is no treatment for *CFAP410*-related retinal dystrophy. Management is conservative and includes appropriate support including low visual aids, assistive technology, social services and support societies as required. A gene therapy for *CFAP410*-related retinal dystrophy would be a good candidate, since the cDNA size of 1.1kb is well within the packaging capacity of the adeno-associated viral vector—the most established vector for retinal gene supplementation (25,26).

In conclusion, we report a novel homozygous in-frame deletion in the *CFAP410* gene with a cone-only dystrophy phenotype without systemic features. This adds to the current genotypic and phenotypic spectra of *CFAP410* related disease, enabling a better understanding which should help inform the development of potential future genetic treatments.
